# Data representing applicability of developed growth hormone 1 (GH1) gene detection method for detecting Atlantic salmon (*Salmo salar*) at high specificity to processed salmon commodities

**DOI:** 10.1016/j.dib.2019.104695

**Published:** 2019-10-22

**Authors:** Keisuke Soga, Kosuke Nakamura, Takumi Ishigaki, Shinya Kimata, Kiyomi Ohmori, Masahiro Kishine, Junichi Mano, Reona Takabatake, Kazumi Kitta, Hiroyuki Nagoya, Kazunari Kondo

**Affiliations:** a National Institute of Health Sciences, 3-25-26 Tonomachi, Kawasaki-ku, Kawasaki, Kanagawa, 215-9501, Japan; b Chemistry Division, Kanagawa Prefectural Institute of Public Health, 1-3-1 Shimomachiya, Chigasaki, Kanagawa, 253-0087, Japan; c Division of Analytical Science, Food Research Institute, National Agriculture and Food Research Organization, 2–1–12 Kannondai, Tsukuba, Ibaraki, 305–8642, Japan; d Research Center for Aquatic Breeding, National Research Institute of Aquaculture, Fisheries Research and Education Agency, 224-1 Hiruta, Tamaki-cho, Mie, 519-0423, Japan

**Keywords:** Specificity, Atlantic salmon, *Salmo salar*, Salmon, Real-time polymerase chain reaction (PCR), Growth hormone 1, Processed foods, Detection method

## Abstract

This article is referred to the research article entitled “Development of a novel method for specific detection of genetically modified Atlantic salmon, AquAdvantage, using real-time polymerase chain reaction” by Soga et al. (2020).

Applicability of the developed *growth hormone 1* (GH1) and *18S ribosomal DNA* (18S rDNA) detection methods using real-time polymerase chain reaction (PCR) for detecting Atlantic salmon (*Salmo salar*) to processed food commodities was examined. DNAs extracted and purified from 24 commodities labelled to include salmon as an ingredient were used as template. Yield and purity of DNAs obtained and Cq values from real-time PCR analyses were provided.

**Table:** Specifications table

Subject	Food Science
Specific subject area	Development of analytical method
Type of data	Tables
How data were acquired	To detect DNA, real-time polymerase chain reaction (PCR) was performed using ABI PRISM 7900 Sequence Detection System (7900HT) (Thermo Fisher Scientific, Waltham, MA, USA). To quantify DNA, ultraviolet (UV) absorption was measured using ND-1000 spectrophotometer (Thermo Fisher Scientific). To extract and purify DNA, GM quicker 3 kit (Nippon gene, Toyama, Japan) and Genomic-tip 20/G (Qiagen, Hilden, Germany) were used. To test applicability of the method, various kinds of salmon commodities produced worldwide were used as test samples.
Data format	Raw
Parameters for data collection	One gram of smoked, sliced, canned and pickled salmon and salmon roe and 0.5 g of dried salmon were used to extract and purify DNA. Fifty nanograms of extracted and purified DNA were used as template for real-time PCR analysis in duplicate tests per a sample. Thermal-cycling conditions were 95 °C for 10 min, followed by 50 cycles of 15 sec at 95 °C and 1 min at 57 °C. SDS 2.4 sequence detection software (Thermo Fisher Scientific) was used to normalize the reporter signal (ΔRn) threshold at 0.2 to calculate cycle threshold (Cq) values.
Description of data collection	DNA samples were quantified by measuring the UV absorption at 260 nm (A260) using ND-1000 spectrophotometer. The quality of the samples was estimated from the UV absorption ratios at 260 and 280 nm (A260/A280) and 260 and 230 nm (A260/A230). For real-time PCR analysis, the baseline was set to cycles 3 through 15. The ΔRn threshold for plotting the cycle threshold (Cq) values was set to 0.2 during exponential amplification. Reactions with the Cq value of less than 48 and exponential amplification plots were scored as positive. If the Cq value could not be obtained, the reaction was scored as negative. Reactions with the Cq value of less than 48, but without exponential amplification as judged by visual inspection of the respective ΔRn plots and multi-component plots were scored as negative.
Data source location	Institution: National Institute of Health Sciences City/Town: Kawasaki/Kanagawa Country: Japan
Data accessibility	With the article
Related research article	Author's name: Keisuke Soga, Kosuke Nakamura, Takumi Ishigaki, Shinya Kimata, Kiyomi Ohmori, Masahiro Kishine, Junichi Mano, Reona Takabatake, Kazumi Kitta, Hiroyuki Nagoya, Kazunari Kondo Title: Development of a novel method for specific detection of genetically modified Atlantic salmon, AquAdvantage, using real-time polymerase chain reaction Journal: Food Chemistry DOI: https://doi.org/10.1016/j.foodchem.2019.125426

**Table:** 

**Value of the Data** • These data provide information concerning applicability of GH1 and 18S rDNA detection methods to various processed salmon commodities to detect *Salmo salar* (Atlantic salmon) ingredient. • The data are beneficial for researchers who are trying to detect or confirm the presence of Atlantic salmon derived ingredient in foods. • These data show potential of developed real-time PCR detection method to monitor Atlantic salmon in various processed salmon commodities at high sensitivity and specificity. • The data provide information concerning DNA yield and purification efficiency from various processed salmon commodities that were produced worldwide and applicability of developed GH1 detection method [1] that was more sensitive than the original method described [2] to the commodities.

## 1 Data

Table 1 summarizes the information on processed salmon commodities, including food processing type, production country, food ingredient labelled in Japan and recommended preservation state. Table 2 is the list of information on sequences of real-time PCR primer pairs and probes used in this dataset. Table 3 presents the specificity test data using developed 18S rDNA detection method. Table 4 shows the data of DNA yields and purity obtained from 24 kinds of processed salmon commodities, and of real-time PCR tests using 18S rDNA and GH1 detection methods.

**Table 1:** Processed salmon commodities used in this dataset.

Sample no.	Food processing type	Commodity name	Production country	Ingredient on the food label^a^ in Japan	Recommended preservation state
1	Smoked	Smoked salmon	Chile	Atlantic salmon, Salt, Sugar	Frozen
2		Smoked salmon	Japan	Chilean cultured Atlantic salmon, Salt, Sugar, Spice	Frozen
3		Smoked salmon	Japan	Norwegian Atlantic salmon, Seaweed salt, White superior soft sugar, Onion powder, (A part of the ingredients contains a salmon.)	Frozen
4	Sliced	Atlantic salmon baked slice	Vietnam	Norwegian cultured Atlantic salmon	Frozen
5		Daily dish	Japan	Atlantic salmon	Frozen
6		Smoked salmon slice	Vietnam	Norwegian Atlantic salmon	Frozen
7	Canned	Atlantic salmon backbone boiled in water	Vietnam	Norwegian Atlantic salmon, Salt	Room temperature
8		Processed salmon	Thailand	Atlantic salmon, Soybean oil, Salt, Garlic paste, Vegetables extract, Spice/Seasoning (Amino acids etc.), Thickener (Guar Gum)	Room temperature
9		Processed fish	France	Atlantic salmon, Food with milk etc., Smoked Atlantic salmon, Wheat flour, Tomato paste, Mustard, Processed milk protein, Salt, Pepper, Allspice, Coriander	Room temperature
10		Salmon midrib boiled in water	Japan	Silver salmon midrib (Oncorhynchus kisutsh), Salt	Room temperature
11		Smoked Atlantic salmon with oil	Japan	Atlantic salmon, Soybean oil, Salt, Sake	Room temperature
12		Pink salmon boiled in water	Japan	Pink salmon (*Oncorhynchus gorbuscha*), Salt	Room temperature
13	Dried	Fishery dried products	Japan	Norwegian Atlantic salmon, Chilean Rainbow trout (*Oncorhynchus mykiss*), Rice vinegar, Seasoning (Amino acids etc.), (A part of the ingredients contains soybean and wheat.)	Room temperature
14		Salmon flakes	Japan	Chum salmon (*Oncorhynchus keta*), Vegetable fats and oils (Rapeseed oil, Soybean oil), Reduced sugar syrup, Salt, Bonito extract powder, Kelp extract/Trehalose, Seasoning (Amino acids etc.), Antioxidant (Vitamin E), Emulsifier, Colorants (Red 102, Yellow 5), (A part of the ingredients contains salmon, soybean and fish sauce.)	Room temperature
15		Salmon flakes	Japan	Chum salmon (*Oncorhynchus keta*), Vegetable fats and oils, Sugar, Salt, Reduced sugar syrup/Seasoning (Inorganic salt etc.), Colorants (Monascus, Carotinoid), (A part of the ingredients contains salmon and soybean.)	Room temperature
16		Pasta sauce	Japan	Salmon sauce (Shortening [Rapeseed oil, Palm oil, Palm kernel oil]), Atlantic Salmon, Dextrin, Salt, Seafood extract, Sugar, Dried bonito extract, Kelp extract, Soy sauce/Seasonings (Amino acids etc.), Thickener (Carrageenan), Flavoring agent, (A part of the ingredients contains wheat, salmon and soybean.), Topping (Laver)	Room temperature
17		Salmon spread	Japan	Natural cheese, Salmon, Smoked salmon, Vegetables paste, Raw cream, Wine, Tomato paste, Spice, Citrus fiber, Garlic saute, Condensed lemon juice/Stabilizer (Locust), Emulsifier, Spice extract, (A part of the ingredients contains milk constituent, salmon and soybean.)	Room temperature
18		Furikake	Japan	Seasoning granule (Lactose, Salt, Salmon powder, Sugar, Salmon extract, Yeast extract), Laver, Salmon flakes (Red salmon, Salmon, Salt, Starch, Vegetable fats and oils, Rice flour, Salmon extract, Lactose, Non-fat soybean, Sugar, Yeast extract), Flakes (Wheat flour, Starch, Salt, Sugar, Vegetable fats and oils)/Seasonings (Amino acids etc.), Carotinoid pigment, Monascus pigment, Antioxidant (Vitamin E), Citric acid	Room temperature
19		Chazuke	Japan	Seasoning granule (Salt, Salmon extract, Sugar, Powdered tea, Seafood extract, Kelp powder), Cracker, Salmon flakes, Laver, Seasoning (Amino acids etc.), Monascus pigment, Antioxidant (Vitamin E), Citric acid	Room temperature
20		Chazuke	Japan	Salmon Chazuke/Salt, Cracker, Sugar, Salmon, Laver, Glucose, Starch, Refined sugar, Powdered tea, Dextrin, Kelp, Fish sauce, Salmon extract, Seasoning (Amino acids etc.), Monascus pigment, Antioxidant (Vitamin E), Acidifier	Room temperature
21	Roe	Salted salmon roe	Japan	Salmon eggs, Salt	Frozen
22		Salted salmon roe	USA	Red salmon eggs, Salt, Seasoning (Amino acid etc.), Antioxidant (Vitamin E), Color former (Sodium nitrous acid)	Frozen
23		Salted salmon roe	Japan	Salmon ovary, Salt, Color former (Sodium nitrous acid)	Frozen
24	Pickled	Salted salmon	Japan	Atlantic salmon, Malted rice, Salmon roe, Sake, Salt, Fermented seasoning, Red pepper, Seasoning (Amino acids etc.), Alcohol, Acidifier, pH Conditioner, Red pepper extract, Stabilizer (Xanthan), Antioxidant (Vitamin C), (A part of the ingredients contains soybean and pork.)	Frozen

a Food label in Japanese was translated in English by authors of this article.

**Table 2:** List of oligonucleotide primers and probes used.

Detection method	Target region (GenBank accession no.)	Name	Nucleotide sequences (5′→3′)^a^	Amplicon (bp)	Reference
GH1	Growth hormone 1 (X61938)	AM5F	AAGGTGCAAAACCATGTTGCCTTCT	176	[1]
		AM5R	ATGTGAGTGTTCTAGGTCACTAGAC		
		AM5PR-2	[FAM]TGCGTTTCTTGTGCTCTCTATTGCAAAGTA[TAMRA]		
18S rDNA	18S ribosomal DNA (FJ710886)	18S–F	TGTGCCGCTAGAGGTGAAATT	61	This dataset
		18S-R	GCAAATGCTTTCGCTTTCG		
		18S–P	[FAM]TTGGACCGGCGCAAGACGG[TAMRA]		

a FAM, 6-carboxyfluorescein; TAMRA, 6-carboxytetramethylrhodamine.

**Table 3:** Specificity test for 18S rDNA real-time PCR method.

	Organism name	NCBI taxonomy ID	Real-time PCR detection^a^	
Plants	*Allium cepa* L.	4679	–	–
	*Apium graveolens* Dulce Group	117781	–	–
	*Arachis hypogaea* L.	3818	–	–
	*Brassica napus* L.	3708	–	–
	*Brassica rapa* var. *perviridis*	344680	–	–
	*Capsicum annuum* var. *annuum*	40321	–	–
	*Carica papaya* L.	3649	–	–
	*Citrus limon* (L.) Burm.f.	2708	–	–
	*Cucurbita* L.	3660	–	–
	*Daucus carota* L.	4039	–	–
	*Glycine max* (L.) Merr.	3847	–	–
	*Oryza sativa* L.	4530	–	–
	*Solanum lycopersicum*	4081	–	–
	*Solanum melongena* L.	4111	–	–
	*Solanum tuberosum* L.	4113	–	–
	*Triticum aestivum* L.	4565	–	–
	*Zea mays* L.	4577	–	–
Animals	*Bos taurus*	9913	40.80	40.56
	*Sus scrofa*	9823	40.54	42.18
Fishes	*Hyperoglyphe japonica*	171196	18.28	18.32
	*Oncorhynchus keta*	8018	19.73	19.63
	*Oncorhynchus kisutch*	8019	19.59	19.48
	*Oncorhynchus masouishikawae*	8021	20.68	20.69
	*Oncorhynchus mykiss*	8022	20.45	20.51
	*Pagrus major*	143350	19.01	19.01
	*Scomber japonicus*	13676	18.70	18.73
	*Salmo salar* (Atlantic salmon)	8030	19.53	19.71
	No template control	–	–	–

–, The reaction was scored as negative.

a Cq values obtained from a duplicate test per a sample were indicated.

**Table 4:** DNA yield and purity obtained from the processed salmon commodities.

Sample No.	Salmon type^a^	DNA yield (ng/g)^b^	DNA purity		Real-time PCR detection^e^			
			A260/A280^c^	A260/A230^d^	18S rDNA method		GH1 method	
1	*Salmo salar* (Atlantic salmon)	87,375	1.53	2.77	17.03	16.90	27.49	27.38
2		95,125	1.56	2.73	16.81	16.71	27.71	27.65
3		63,875	1.50	2.79	17.03	16.99	27.22	27.12
4		64,625	1.50	2.77	16.51	16.45	27.13	27.08
5		126,600	1.53	2.68	16.99	16.96	27.60	27.53
6		101,800	1.55	2.61	17.12	16.99	27.90	28.03
7		40,700	1.56	2.48	19.96	20.01	35.12	35.92
8		84,400	1.52	2.64	18.98	18.99	31.16	31.27
9		48,250	1.49	2.90	19.05	19.16	32.48	32.63
11		158,250	1.52	2.72	19.42	19.45	32.92	32.92
13		325,000	1.45	2.70	19.55	19.49	31.35	31.50
16		38,750	1.48	2.99	18.06	17.97	27.42	27.37
24		89,500	1.62	2.64	18.44	18.89	28.35	28.79
10	*Oncorhynchus kisutsh*	68,250	1.46	3.05	21.24	21.30	–	–
12	*Oncorhynchus gorbuscha*	171,250	1.53	2.70	19.34	19.29	–	–
14	*Oncorhynchus keta*	143,500	1.46	2.79	19.26	19.11	–	–
15		146,000	1.45	2.76	21.03	20.84	–	–
22	*Oncorhynchus nerka*	47,200	1.56	2.64	19.30	19.12	–	–
17	Unknown	2,063	1.51	1.33	24.76	24.85	–	42.14
18		56,400	1.49	2.21	20.53	20.22	–	–
19		84,000	1.55	2.62	19.00	18.80	–	–
20		20,400	1.47	2.64	20.28	20.43	–	–
21		20,500	1.45	2.71	19.42	19.43	–	–
23		16,200	1.49	2.63	22.66	22.39	–	–

–, The reaction was scored as negative.

a According to the information on food label.

b DNA yield (ng) per 1 g sample.

c Absorbance ratio at 260 and 280 nm.

d Absorbance ratio at 260 and 230 nm.

e Cq values obtained from a duplicate test per a sample were indicated.

## 2 Experimental design, materials, and methods

Twenty-four processed salmon commodities including six types (smoked, sliced, canned, dried, roe and pickled) were purchased through the Internet in Japan. Details of food ingredients labelled, production country and recommended preservation state were described in Table 1. The commodities were selected taking into account that a wide range of salmonids species is covered in the test, based on the phylogeny identified in salmonid species [3].

Sampling weight was 1 g for smoked, sliced, canned, roe and pickled commodities, and 0.5 g for dried commodities. DNA extraction and purification were done using GM quicker 3 kit (Nippon gene, Toyama, Japan) for smoked, sliced, canned and dried commodities. Genomic-tip 20/G (Qiagen, Hilden, Germany) was used for salmon roe and pickled commodities. The quantity and the quality of DNA obtained were estimated from the ultraviolet (UV) absorption at 260 nm and its ratios at 260 and 280 nm (A260/A280) and 260 and 230 nm (A260/A230), respectively, using ND-1000 spectrophotometer (Thermo Fisher Scientific).

The primer pair and probe targeting GH1 were used as described previously [1,2]. The primer pair and probe targeting 18S rDNA were designed using Primer Express Software (Thermo Fisher Scientific, Version 3.0.1), based on the sequence given by NCBI (*Salmo salar* 18S ribosomal RNA gene, partial sequence, GenBank accession no. FJ710886) (Table 2). The probes were labelled with 6-carboxyfluorescein (FAM) at 5′ terminus and with 6-carboxytetramethylrhodamine (TAMRA) at 3’ terminus. Oligonucleotide sequences of primer pairs and probes used in this dataset are shown in Table 2.

Fifty nanograms of extracted and purified DNA were used as template for real-time PCR analysis in duplicate tests per a sample. The fluorescence intensity when amplifying targeted DNA sequences was monitored by ABI PRISM 7900 Sequence Detection System (Thermo Fisher Scientific). Thermal cycling conditions were 95 °C for 10 min, followed by 50 cycles of 15 sec at 95 °C and 1 min at 57 °C.The baseline was set to cycles 3 through 15. The ΔRn threshold for plotting the cycle threshold (Cq) values was set to 0.2 during exponential amplification.

For tests, reactions with the Cq value of less than 48 and exponential amplification plots were scored as positive. If the Cq value was more than 48 or could not be obtained, the reaction was scored as negative. Reactions with the Cq value of less than 48, but without exponential amplification as judged by visual inspection of the respective ΔRn plots and multi-component plots were scored as negative. Specificity test of 18S rDNA method was performed using 17 kinds of plants, 2 kinds of animals and 8 kinds of fishes (Table 3).
